# Effect of electroacupuncture on the intestinal microflora in rats with stress urinary incontinence

**DOI:** 10.3389/fendo.2022.860100

**Published:** 2022-07-29

**Authors:** Chaonan Li, Zhiyu Qu, Jiandang Liu, Shuoquan Ruan, Bingli Chen, Jinchuan Ran, Wen Shu, Yuelai Chen, Wenguang Hou

**Affiliations:** Yueyang Hospital of Integrated Traditional Chinese and Western Medicine, Shanghai University of Traditional Chinese Medicine, Shanghai, China

**Keywords:** electroacupuncture, stress urinary incontinence, urodynamics, intestinal flora, blautia

## Abstract

**Objective:**

To examine the effect of electroacupuncture on the urodynamics and gut microbiota of rats with stress urinary incontinence (SUI).

**Materials and methods:**

Thirty 2-month-old female Sprague–Dawley (SD) rats were randomly assigned to 4 groups: normal (N), model (M), nonacupoint electric acupuncture control (NAAC), and electroacupuncture (EA). An SUI rat model was established through vaginal balloon dilatation and bilateral oophorectomy. After various treatments, urodynamic tests were performed, and feces were collected. 16S rRNA sequencing analysis was used to investigate SUI-related changes in the intestinal flora.

**Results:**

After treatment, compared with those of the M group, the leak point pressure and maximum bladder capacity of the electroacupuncture groups increased (*P*<0.05). The species community compositions of the N and M groups differed at the genus level, and there were 15 differentially abundant bacterial genera (*P*<0.05). The Blautia proportion was increased by electroacupuncture treatment (*P*<0.05) and was significantly positively correlated with the electroacupuncture treatment of SUI (according to Spearman correlation analysis).

**Conclusion:**

Electroacupuncture treatment can improve signs of urine leakage in rats with SUI rats by increasing the leak point pressure and maximum bladder capacity. The enrichment of Blautia by electroacupuncture treatment enrichment may be related to SUI sign improvement.

## Introduction

Stress urinary incontinence (SUI) often occurs in middle-aged and elderly women. When abdominal pressure increases, involuntary urine leakage occurs (1). SUI is closely related to vaginal delivery. Vaginal delivery is one of the main causes of SUI and can affect the pelvic muscles, nerves and connective tissues, resulting in insufficient bladder neck and urethral support (2), external urethral sphincter (EUS) and pudendal nerve injury (3). If the endopelvic fascia of the urethra and bladder fascia and the anterior wall of the vagina are damaged, the force of the vagina compressing the urethra will be weakened, resulting in the involuntary outflow of urine when the abdominal pressure increases. EUS and pudendal nerve injury result in EUS weakness and involuntary leakage of urine. Electrical stimulation of the pudendal nerve can promote nerve regeneration and function in rats with SUI (4). The lack of estrogen after menopause changes the metabolism of connective tissue and reduces the production of collagen, which may lead to the occurrence of SUI (5). Studies have shown that estrogen receptors are located in the vagina, urethra, bladder and pelvic floor muscle tissue. The lack of estrogen after menopause is related to the thinning of the submucosa and atrophic changes in the pelvic floor myofascial structure. Atrophic changes in the structure lead to a decrease in the closure pressure of the urethra, which in turn promotes the occurrence of SUI (6, 7). Bladder dysfunction and bowel dysfunction commonly cooccur in clinical practice. Other studies have shown that anal sphincter tears caused by levator ani injury during vaginal delivery are closely related to the development of fecal incontinence (FI) (8). The pudendal nerve also innervates the external anal sphincter. Lower urinary tract symptoms and FI sometimes occur concurrently (9). Electrical stimulation of the pudendal nerve can increase the pressure of the anal sphincter and improve FI symptoms (10).

Some data from experimental animal and human studies have shown that lower urinary tract and intestinal disorders usually occur at the same time (11). A healthy intestinal microbiota can promote individual nutritional metabolism, intestinal mucosal growth and other functions and is related to individual health (12). For example, microbial populations that reside in experimental animals may influence bladder cancer development and/or treatment response (13). Acupuncture, a traditional Chinese therapeutic approach, allows nonpharmaceutical treatment of SUI. In recent years, electroacupuncture (EA) stimulation of the lumbosacral region has been shown to reduce the frequency and extent of urine leakage in women with SUI (14). In this study, female SD rats were used to establish an SUI model and perform intravaginal balloon dilatation (similar to birth injury) combined with oophorectomy (simulated menopause). EA was used to stimulate acupuncture points as an intervention method. We observed the therapeutic effect of EA on SUI through urodynamic measurements, collected stool samples for 16S rRNA high-throughput sequencing to analyze the changes in intestinal flora and explored whether there was a relationship between the changes in intestinal flora and EA treatment of SUI.

## Materials and methods

### Animals and study design

Thirty normal healthy female SD rats weighing 220-250 g were purchased from Beijing Vital River Laboratory Animal Technology Co., Ltd. All rats were provided free access to food and water, maintained under a 12-h light/dark cycle, and randomly assigned to 4 groups: N (n = 6), M (n = 8), NAAC (n = 8), and EA (n = 8). All rats except the N group underwent vaginal balloon dilatation and bilateral oophorectomy to replicate the SUI model (15). Briefly, after anesthesia (sodium pentobarbital, 30 mg/kg, i.p.), an 8-Fr latex Foley catheter was inserted into the rat’s vagina, and 4.0 ml of sterile saline was slowly pushed into the balloon to expand the vagina. The catheter was sutured and fixed, and a 120 g weight was suspended in the drooping injection port. The catheter was removed after 4 hours of traction, and both ovaries were excised with a midline incision after establishing the model.

In the experiment, two urodynamic measurements were made (to reduce the discomfort of the rats, the measurements were made after anesthesia): one week after modeling and one day after the end of treatment. After emptying the bladder, a 0.7 mm epidural catheter was inserted 2 cm into the rat bladder before fixing it. One end was connected to a microinjection pump through a three-way connector to the bladder, and sterile saline was injected at a speed of 0.3 ml/min. One end was connected to a urodynamic testing instrument (Bonito urodynamic detector, Laborie Medical Technology Company of Canada). The urodynamic testing instrument was calibrated to “0”, and the test was started. When the first drop of fluid appeared at the urethral orifice, perfusion was stopped. At this time, the bladder capacity (perfusion time multiplied by perfusion speed) and intravesical pressures were recorded to determine the leak point pressure (LPP) and maximum bladder capacity (MBC). The sneeze test was carried out after the first urodynamic measurement (16). The success models (n = 17) were randomly assigned to 3 groups: the M (n = 5), NAAC (n = 6), and EA (n = 6) groups.

### Treatment protocol

After the first urodynamic measurement, the N and M groups did not receive any treatment. The EA group was treated by electrical stimulation (density wave, 4/20 Hz, 20 min) at the acupoints of Shenshu (BL23, on both sides of the spinous process on the lower back) and Huiyang (BL35, positioned almost vertically underneath the periosteum approximately 5 mm lateral to the midline of the coccyx, bilateral symmetry), determined relative to their anatomical locations described in the WHO guidelines for human acupoints (17). A disposable acupuncture needle (diameter, 0.25 mm) was connected to an SDZ-V EA instrument (Shanghai, China). EA treatment was performed once a day and continued for 3 d. For the NAAC group, stimulation was at the Shenshu control point (1 cm beside Shenshu) and at the Huiyang control point (1 cm beside Huiyang). The stimulation settings were the same as in the EA group. The second urodynamic measurements were made after the above treatments. Rat stool samples were collected and stored, and 16S rRNA sequencing was performed to analyze the changes in SUI-related intestinal flora.

### Dna extraction and Pcr amplification

After the experiment, 1-2 stool samples each from 4 rats randomly selected from each group were collected using the Omega kit (Omega Bio-Tek, Norcross, GA, U.S.). For DNA extraction, a NanoDrop 2000 was used to assess the DNA purity and concentration, and agarose gel electrophoresis was used to assess DNA integrity. The primers 338F (5’-ACTCCTACGGGAGGCAGCAG-3’) and 806R (5’-GGACTACHVGGGTWTCTAAT-3’) were used for PCR amplification of the V3-V4 variable region of 16S rRNA as follows: denaturing at 95°C for 30 s, annealing at 55°C for 30 s, and extension at 72°C for 30 s for 27 cycles, and a final extension at 72°C for 10 min (PCR instrument: ABGeneAm p^®^ 9700). The amplification reaction consisted of 4 µl of 5* Fast Pfu buffer, 2 µl of dNTPs at 2.5 mM, 0.8 µl of primer (5 µM), 4 µl of Fast Pfu polymerase, and 10 ng of DNA template.

### Illumina Miseq sequencing

Samples were sequenced and analyzed on an Illumina MiSeq platform (Illumina, San Diego, USA) according to the standard protocols by Majorbio Bio-Pharm Technology Co., Ltd. (Shanghai, China). The raw reads were quality controlled with Trimmomatic software, and FLASH software was used for read assembly. Operational taxonomic units (OTUs) were identified at a 97% similarity cutoff using UPARSE (version 7.1, http://drive5.com/uparse/), and chimeric sequences were identified and removed. The taxonomy of each representative OTU sequence was analyzed by RDP Classifier (http://rdp.cme.msu.edu/) against the 16S rRNA database (Silva SSU132) using a confidence threshold of 0.7.

### Data analysis

Data were analyzed with GraphPad Prism 6.0. One–way analysis of variance (ANOVA) was used to assess the difference between groups, followed by pairwise comparison using the least significant difference (LSD) test. *P*<0.05 indicated that the difference was statistically significant. The measured data are expressed as the mean ± SEM. The rarefaction curves and alpha diversity indices referring to community richness (Chao1) and community diversity (Shannon) were based on the OTU information. The species composition is represented in a histogram. Diversity among groups was shown by the phylogenetic beta diversities calculated by partial least squares discriminant analysis (PLS-DA) with unweighted UniFrac distances. ANOSIM/Adonis analysis was used to determine whether the grouping was meaningful by testing if the difference between groups was significantly greater than those within groups. Based on a normalized relative abundance matrix, the linear discriminant analysis (LDA) effect size (LEfSe) method based on the Kruskal–Wallis test was used to identify potential biomarkers. The LDA threshold was 2.0, and the significance α was 0.05.

## Results

### EA improves LPP and MBC in rats with SUI

**Figure 1:**
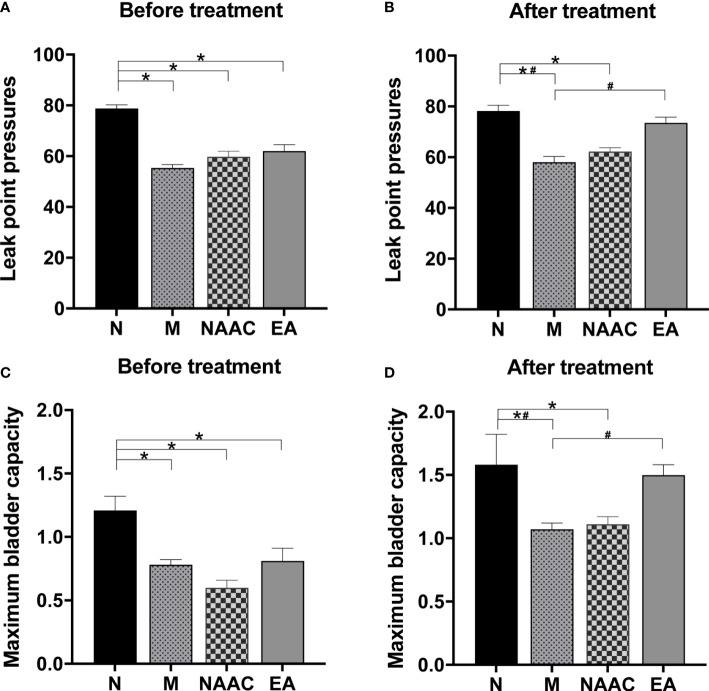
The effects of EA on LPP **(A, B)** and MBC **(C, D)** in rats with SUI (N=6, M=5, NAAC=6, EA=6). **(A)** The LPP before treatment was analyzed *via* one-way analysis of variance (ANOVA) (F (3,19) = 27.702, *P*=0.001 <0.05), followed by pairwise comparison using the least significant difference (LSD) test. **(B)** The LPP after treatment was analyzed *via* ANOVA (F (3,19) = 19.551, *P*=0.001 <0.05), followed by pairwise comparison using the LSD test. **(C)** The MBC before treatment was analyzed *via* ANOVA (F (3,19) = 8.515, *P*=0.001 <0.05), followed by pairwise comparison using the LSD test. **(D)** The MBC after treatment was analyzed *via* ANOVA (F (3,19) = 3.455, *P*=0.037 <0.05), followed by pairwise comparison using the LSD test. *P < 0.05 compared with the N group; ^#^ *P* < 0.05 compared with the M group.

The effect of EA on LPP in rats with SUI is shown in **Figures 1A**, **B**. Before treatment (**Figure 1A**), compared with that of the N group (78.83 ± 1.40), the LPPs of the M (55.40 ± 1.29), NAAC (59.83 ± 2.13), and EA groups (62.00 ± 2.50) decreased (*P <*0.05). After treatment (**Figure 1B**), the LPP of the M group (58.20 ± 2.15) was significantly lower than that of the N group (78.17 ± 2.27) (*P <*0.05), and the LPP of the rats with SUI increased significantly after EA treatment (73.50 ± 2.32) (*P <*0.05), while there was no significant difference between the NAAC group (62.17 ± 1.54) and the M group (*P* =0.607>0.05). Similarly, the effect of EA on MBC in rats with SUI is shown in **Figures 1C**, **D**. Before treatment (**Figure 1C**), compared with that of the N group (1.21 ± 0.11), the MBCs of the M (0.76 ± 0.07), NAAC (0.60 ± 0.06), and EA groups (0.81 ± 0.10) decreased (*P <*0.05). After treatment (**Figure 1D**), the MBC of the M group (1.08 ± 0.05) was significantly lower than that of the N group (1.58 ± 0.24) (*P <*0.05), and the MBC of the rats with SUI increased significantly after EA treatment (1.50 ± 0.08) (*P <*0.05), while there was no significant difference between the NAAC (1.11 ± 0.06) group and the M group (*P* =1.000>0.05).

### Diversity of the microbial community associated with the rats in each group

**Figure 2:**
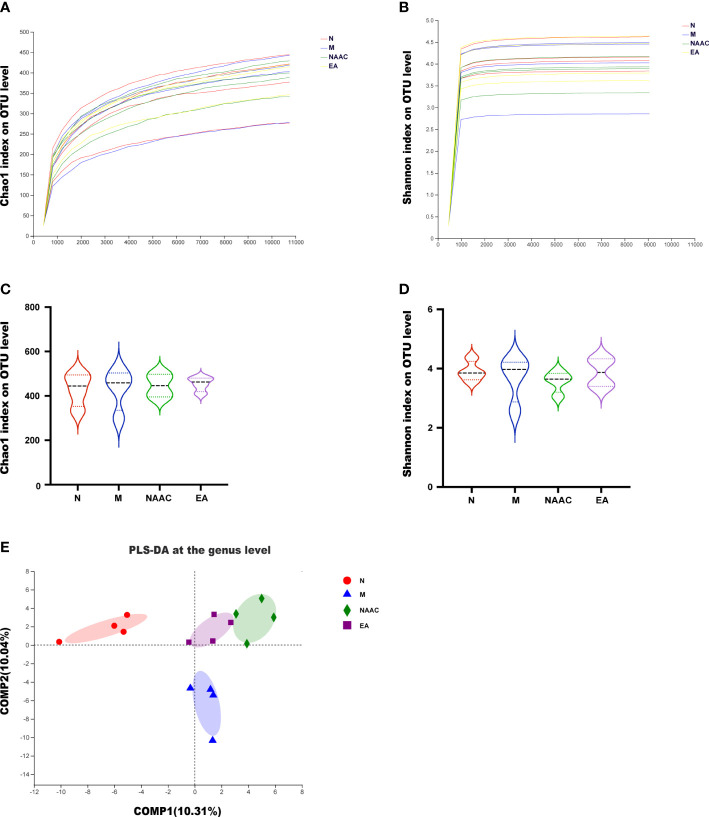
Diversity of the microbial community associated with the rats in each group. Alpha rarefaction curve **(A, B)** and violin plot analysis **(C, D)**. The alpha index rarefaction curves show the differences in species abundance among samples and evaluate the rationality of the sequencing quantity of samples. Violin plot analysis of alpha diversity using the Kruskal–Wallis test reflected the dispersion degree of samples in the group and the intergroup index differences. The Chao1 index **(A, C)** reflected richness, and the Shannon **(B, D)** index considered diversity. **(E)** Beta diversity (PLS-DA) of the four groups, showing the similarities and differences in species composition among different individuals or groups. The results demonstrated that there were differences in the composition of the four groups.

MiSeq high-throughput sequencing was used to analyze 4 groups of rat stool samples. The alpha exponential rarefaction curve of the Chao1 index (**Figure 2A**) and the Shannon index (**Figure 2B**) showed clear asymptotes, which indicated near-complete sampling of the community. Violin plots of the Chao index (**Figure 2C**)

and the Shannon index (**Figure 2D**) exhibited no significant differences among the four groups. According to ANOSIM/Adonis analysis (R = 0.4721, *P* = 0.001) and PLS-DA (**Figure 2E**), there was a significant difference in the species community composition among the groups.

### Composition of rat microbial flora between the treatment groups

**Figure 3:**
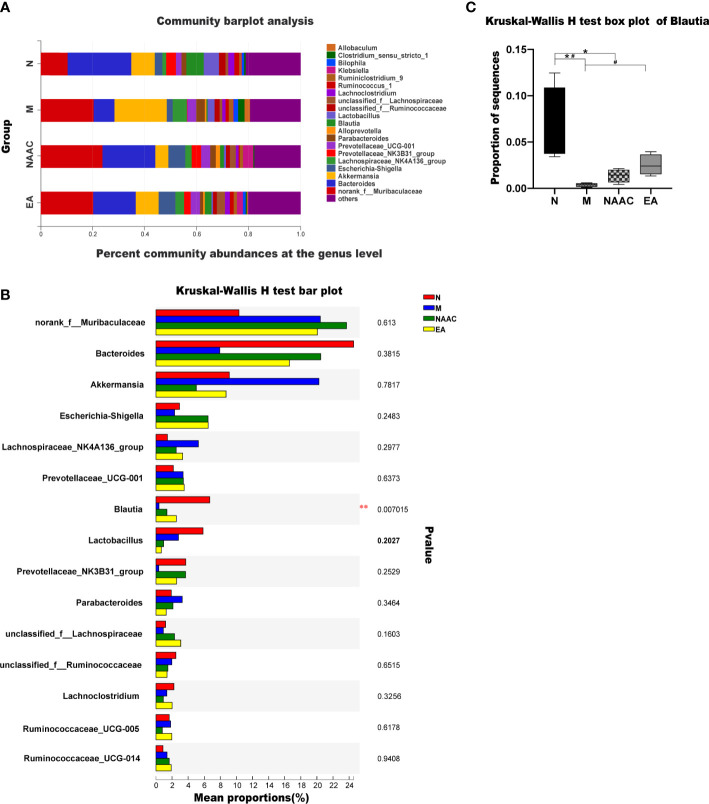
Community bar plot analysis showing the flora community composition at the genus level. The dominant bacteria in the N group and the EA group were the same, namely, Bacteroides and norank_f_Muribaculaceae **(A)**. The Kruskal–Wallis rank-sum test bar plot shows the top 15 species with the most abundant expression at the genus level **(B)**. *0.01< P <=0.05, **0.001< P <=0.01. The relative abundance of Blautia in each group was analyzed via the Kruskal–Wallis test (H = 12.110, P=0.007, <0.05) **(C)**. *P < 0.05 compared with the N group; ^#^P < 0.05 compared with the M group.

**Figure 4:**
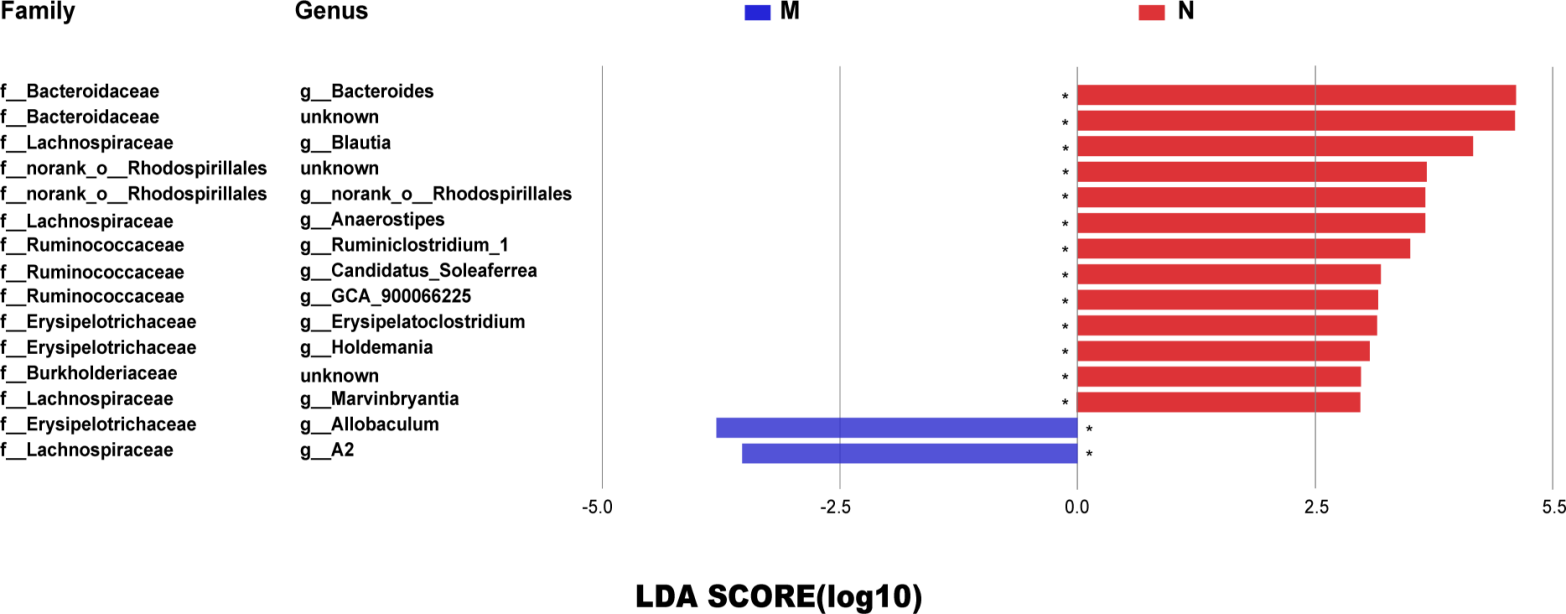
The LDA score identified the size differentiation between the N and M groups with a threshold value of 2. *0.01< *P* ≤0.05. The relative abundances of the Allobaculum and A2 genera in the M group increased, while the relative abundances of Bacteroides, Bacteroidaceae, Blautia, f_norank_o_Rhodospirillales, norank_o_Rhodospirillales Anaerostipes, g_Ruminiclostridium_1, g_Candidatus_Soleaferrea, g_GCA_900066225, Erysipelatoclostridium, Holdemania, Burkholderiaceae, and g_Marvinbryantia decreased (LDA = 2.0, *P <*0.05).

**Figure 3A** shows the OTU clustering results detailing the relative abundances of the bacterial community at the genus level in each group of rat stool samples. We found that the dominant bacteria in the N group and the EA group were the same, namely, Bacteroides and norank_f_Muribaculaceae. However, the mean proportions were different. **Figure 3B** shows the top 15 species with the most abundant expression at the genus level, in which the relative abundance of blautia is statistically different among groups (*0.01< P < =0.05, **0.001< P < =0.01), The comparison of the relative abundance of blautia in rats of each group is shown in **Figure 3C**. Blautia accounted for 6.68%, 0.37%, 1.36% and 2.52% of the microbial communities of the N group, M group, NAAC group and EA group,respectively, indicating that the content of Blautia increased after EA treatment.Electroacupuncture stimulation can significantly increase or decrease the relative abundance of blautia in Sui rats, but electroacupuncture control group can not reverse this change.Further analysis of 15 different bacteria in the N and M groups at different taxonomic levels by LEfSe (**Figure 4**) revealed that the relative abundances of the Allobaculum and A2 genera in the M group increased, while the relative abundances of Bacteroides, Bacteroidaceae, Blautia, f_norank_o_Rhodospirillales, norank_o_Rhodospirillales Anaerostipes, g_Ruminiclostridium_1, g_Candidatus_ Soleaferrea, g_GCA_900066225, Erysipelatoclostridium, Holdemania, Burkholderiaceae, and g_Marvinbryantia decreased (LDA = 2.0, P<0.05).

### The relationship between microbial flora and urodynamics in rats

**Figure 5:**
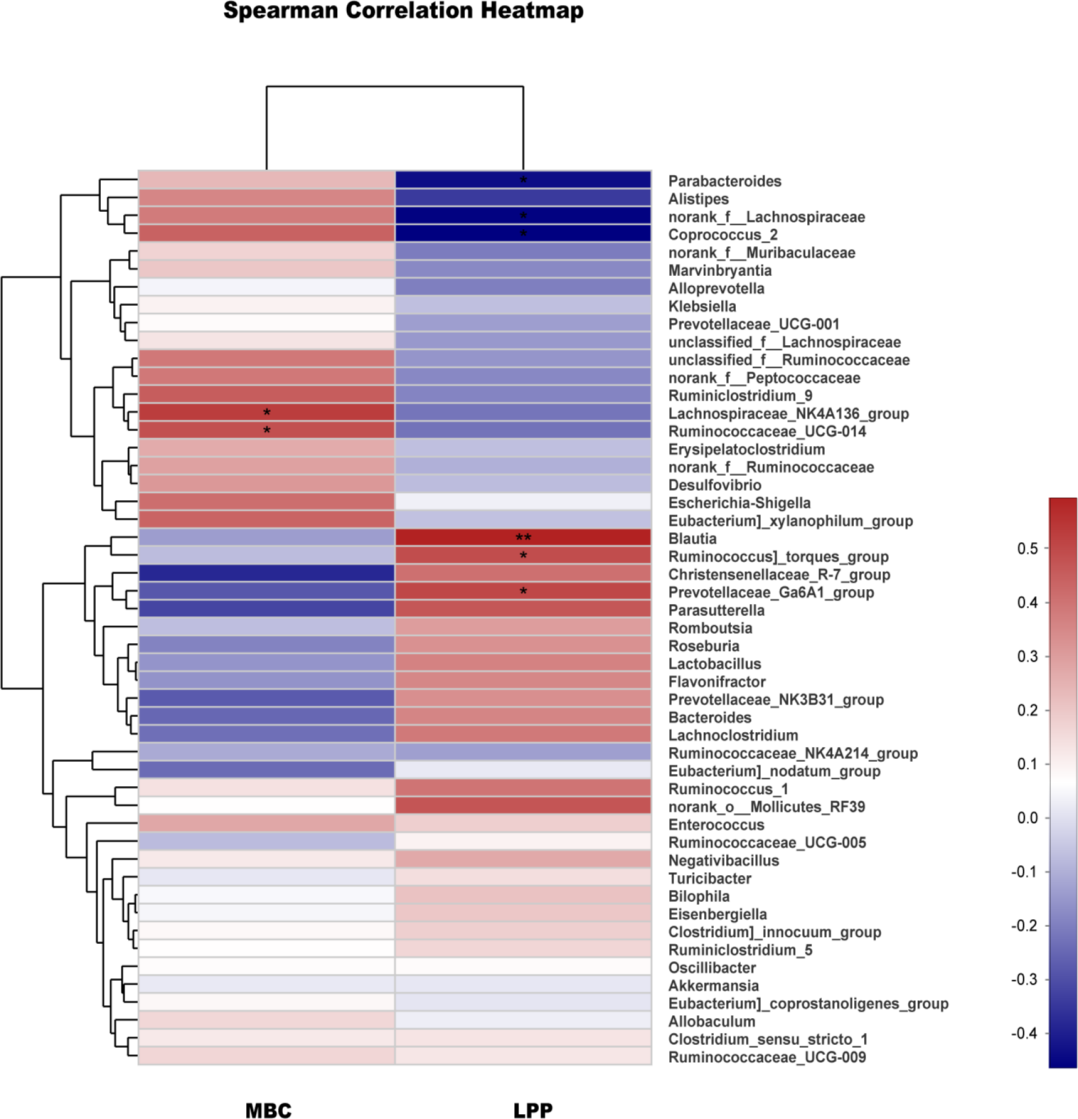
Correlation analysis between urodynamics and different genera. Correlation analysis between MBC, LPP and the major genera. The analysis was conducted using Spearman’s test. Red represents a positive correlation, and blue represents a negative correlation; the deeper the color is, the stronger the correlation (*0.01< P ≤0.05, **0.001< P ≤0.01).

Spearman correlation analysis of a heatmap showed the relationship between microbial flora and urodynamics (**Figure 5**). The bacterial genera positively correlated with MBC were the Lachnospiraceae NK4A136 group and Ruminococcaceae_UCG-014. The bacterial genera positively corelated with LPP were Blautia, Ruminococcus_torques_group and Prevotellaceae_Ga6A1_group, while Parabacteroides, norank_f_Lachnospiraceae and Coprococcus_2 were negatively correlated with LPP. The results of Spearman correlation analysis for the N, M, and EA groups are shown in **Table 1**. The treatment of SUI rats in the EA group was positively correlated with the relative abundance of the genera Blautia and Prevotellaceae_NK3B31_group in terms of the MBC, LPP and sneezing experiments and negatively correlated with the relative abundance of Parabacteroides.

**Table 1:** Correlation coefficients between acupuncture treatment and differential bacteria at the family level, *0.01< P ≤0.05, **0.001< P ≤0.01, *** P ≤0.001.

Bacteria	Correlation coefficient
Blautia	0.856***
Prevotellaceae_ NK3B31_group	0.709**
Parabacteroides	-0.760**

## Discussion

The pathogenesis of SUI is mainly related to an increase in the amount of matrix metalloproteinase-degraded collagen in the extracellular matrix of the pelvic floor support tissues, which weakens the muscle support of the urethra and urethral supporting tissues, resulting in the involuntary outflow of urine when the abdominal pressure increases (18–20). Some studies have shown that increased expression of matrix metalloproteinases is accompanied by intestinal inflammation, and there is a relationship between the composition of the microbiome in the intestine and muscle mass (21, 22). Our previous studies have shown that electroacupuncture can improve the degree of SUI by slowing down the degradation of collagen in the anterior vaginal wall of pelvic floor supporting tissue in SUI rats (23). Our study found that EA treatment changed the species composition of the intestinal flora in rats with SUI. Whether there is a link between the influence of EA on intestinal flora and the regulation of collagen metabolism is still unknown.

Aerobic exercise can increase the relative abundance of Bacteroides in the intestinal flora of menopausal women. The increase in Bacteroides abundance is related to the improvement in blood sugar and lipid metabolism and is positively correlated with cardiopulmonary function (24, 25). Bacteria maintain a complex and generally beneficial relationship with the host when retained in the gut, but when they escape this environment, they can cause different degrees of pathological changes. On the one hand, Bacteroides, as a symbiont, has the following functions: (1) decomposition of complex polysaccharides through a starch utilization system, (2) T-cell activation *via* zwitterionic polysaccharides, and (3) prevention of pathogen colonization in the gastrointestinal tract (26). On the other hand, Bacteroides, as a pathogen, includes the Bacteroides protein BVU, the N-terminal domains 4064 and bf1687 of which may participate in adhesion interactions to mediate multiple actions on the cell surface or extracellular matrix (27). For example, *Bacteroides fragilis* can not only bind to type I collagen through the collagen binding protein (CBP1) gene encoding adhesive collagen (28) but also convert plasminogen in plasma into plasmin through the outer membrane protein bfp60 to increase the degradation of fibrin and collagen (29). We found that the proportion of Blautia in rats with SUI increased after EA treatment, and the relative abundance of Blautia was positively correlated with the change in LPP after EA. Blautia species are thought to be important players in human health based on the correlation between Blautia abundance and various health conditions, including diminished abundance in the elderly (30) and in patients with colorectal cancer (31). Blautia abundance was found to be negatively correlated with visceral fat accumulation in adults (32).

In conclusion, EA at the “Shenshu” and “Huiyang” points can increase LPP and MBC in rats with SUI. We found that multiple bacterial genera in the intestinal microbial genera of rats differed between the N group and the M group. Among them, Blautia was significantly positively correlated with the effect of EA treatment of SUI. However, whether the effect of EA on the intestinal microflora of rats with SUI is related to the decrease in collagen degradation remains to be clarified in future studies.

## Data availability statement

The datasets presented in this study can be found in online repositories. The names of the repository/repositories and accession number(s) can be found below: https://www.ncbi.nlm.nih.gov/, SRP287916.

## Ethics statement

The entire study was carried out upon approval by the animal research ethics committee of Shanghai University of TCM (No. PZSHUTCM 18122110).

## Author contributions

CL, ZQ, JL and SR conducted the research experiments. CL and BC wrote the manuscript and prepared the figures. CL, JR, Wen Shu analyzed the data. YC and WH designed the research and provided oversight of manuscript writing. All authors contributed to the article and approved the submitted version.

## Funding

This study was supported by the National Natural Science Foundation of China (No. 81774409, 81674090).

## Acknowledgments

This study was supported by the National Natural Science Foundation of China (No. 81774409, 81674090).

## Conflict of interest

The authors declare that the research was conducted in the absence of any commercial or financial relationships that could be construed as a potential conflict of interest.

## Publisher’s note

All claims expressed in this article are solely those of the authors and do not necessarily represent those of their affiliated organizations, or those of the publisher, the editors and the reviewers. Any product that may be evaluated in this article, or claim that may be made by its manufacturer, is not guaranteed or endorsed by the publisher.

## References

[1] D'Ancona C Haylen B Oelke M Abranches-Monteiro L Arnold E Goldman H. The international continence society (ICS) report on the terminology for adult male lower urinary tract and pelvic floor symptoms and dysfunction. Neurourol Urodyn (2019) 38:433–77. doi: 10.1002/nau.23897 30681183

[2] Cavalcanti GA Manzano GM Nunes KF Giuliano LM de Menezes TA Bruschini H. Electrophysiological evaluation of the pudendal nerve and urethral innervation in female stress urinary incontinence. Int Urogynecol J (2013) 24(5):801–7. doi: 10.1007/s00192-012-1931-8 22961497

[3] Snooks SJ Setchell M Swash M Henry MM. Injury to innervation of pelvic floor sphincter musculature in childbirth. Lancet (1984) 2(8402):546–50. doi: 10.1016/s0140-6736(84)90766-9 6147604

[4] Jiang HH Song QX Gill BC Balog BM Juarez R Cruz Y. Electrical stimulation of the pudendal nerve promotes neuroregeneration and functional recovery from stress urinary incontinence in a rat model. Am J Physiol Renal Physiol (2018) 315(6):F1555–64. doi: 10.1152/ajprenal.00431.2017 PMC6336991 30132345

[5] Frani D Fistoni I. Laser therapy in the treatment of female urinary incontinence and genitourinary syndrome of menopause: An update. BioMed Res Int (2019) 1–9. doi: 10.1155/2019/1576359 PMC6582847 31275962

[6] Robinson D Toozs-Hobson P Cardozo L. The effect of hormones on the lower urinary tract. Menopause Int (2013) 19(4):155–62. doi: 10.1177/1754045313511398 24336244

[7] Blakeman PJ Hilton P Bulmer JN. Cellular proliferation in the female lower urinary tract with reference to oestrogen status. Bjog Int J Obstetrics Gynaecol (2001) 108(8):813–6. doi: 10.1111/j.1471-0528.2001.00210.x 11510705

[8] Oberwalder M Connor J Wexner SD. Meta-analysis to determine the incidence of obstetric anal sphincter damage. Br J Surg (2003) 90(11):1333–7. doi: 10.1002/bjs.4369 14598410

[9] Manning J Eyers AA Korda A Benness C Michael J Solomon . Is there an association between faecal incontinence and lower urinary dysfunction? Dis Colon Rectum (2001) 44(6):790–8. doi: 10.1007/BF02234696 11391137

[10] Damaser MS Salcedo L Wang G Zaszczurynski P Cruz MA Butler RS. Electrical stimulation of anal sphincter or pudendal nerve improves anal sphincter pressure. Dis Colon Rectum (2012) 55(12):1284–94. doi: 10.1097/DCR.0b013e31826ae2f8 23135588

[11] Panicker JN Marcelissen T von Gontard A Vrijens D Abrams P Wyndaele M. Bladder-bowel interactions: Do we understand pelvic organ cross: ensitization? international consultation on incontinence research society (ICI-RS) 2018. Neurourol Urodynamics (2019) 38(S5):S25–34. doi: 10.1002/nau.24111 31821639

[12] Jandhyala SM Talukdar R Subramanyam C Vuyyuru H Sasikala M Nageshwar Reddy D. Role of the normal gut microbiota. World J Gastroenterol (2015) 21(29):8787–803. doi: 10.3748/wjg.v21.i29.8787 PMC4528021 26269668

[13] He C Huang L Lei P. Sulforaphane normalizes intestinal flora and enhances gut barrier in mice with bbn-induced bladder cancer. Mol Nutr Food Res (2018) 62(24):e1800427. doi: 10.1002/mnfr.201800427 30302904

[14] He C Huang L Lei P Liu X Li B Shan Y. Effect of electroacupuncture on urinary leakage among women with stress urinary incontinence: A randomized clinical trial. JAMA (2017) 317(24):2493–501. doi: 10.1001/jama.2017.7220 PMC5815072 28655016

[15] Resplande J Gholami SS Graziottin TM Rogers R Lin CS Leng W. Long-term effect of ovariectomy and simulated birth trauma on the lower urinary tract of female rats. J Urol (2002) 168:323–30. doi: 10.1097/00005392-200207000-00099 12050564

[16] Kaiho Y Kamo I Chancellor MB Arai Y de Groat WC Yoshimura N. Role of noradrenergic pathways in sneeze-induced urethral continence reflex in rats. Am J Physiol Renal Physiol (2007) 292(2):F639–46. doi: 10.1152/ajprenal.00226.2006 17047168

[17] World Health Organization. WHO Standard Acupuncture Point Locations in the Western Pacific Region. (Manila, Philippines: WHO Western Pacific Region) (2008).

[18] Min J Li B Liu C Guo W Hong S Tang J. Extracellular matrix metabolism disorder induced by mechanical strain on human parametrial ligament fibroblasts. Mol Med Rep (2017) 15(5):3278–84. doi: 10.3892/mmr.2017.6372 28339064

[19] Rahn DD Acevedo JF Word RA. Effect of vaginal distention on elastic fibre synthesis and matrix degradation in the vaginal wall: potential role in the pathogenesis of pelvic organ prolapse. Am J Physiol Regul Integr Comp Physiol (2008) 295(4):R1351–8. doi: 10.1152/ajpregu.90447.2008 PMC3774207 18635445

[20] Sangsawang B Sangsawang N. Stress urinary incontinence in pregnant women: a review of prevalence, pathophysiology, and treatment. Int Urogynecol J (2013) 24(6):901–12. doi: 10.1007/s00192-013-2061-7 PMC3671107 23436035

[21] Heimesaat MM Dunay IR Fuchs D Trautmann D Fischer A Kühl AA. Selective gelatinase blockage ameliorates acute DSS colitis. Eur J Microbiol Immunol (Bp) (2011) 1(3):228–36. doi: 10.1556/EuJMI.1.2011.3.7 PMC3906619 24516729

[22] Siddharth J Chakrabarti A Pannérec A Karaz S Morin-Rivron D Masoodi M. Ageing and sarcopenia associate with specific interactions between gut microbes, serum biomarkers and host physiology in rats. Ageing (Albany NY) (2017) 9:1698–720. doi: 10.18632/ageing.101262 PMC5559170 28783713

[23] Li C Yang M Qu Z Ruan S Chen B Ran J. Effect of electroacupuncture on the degradation of collagen in pelvic floor supporting tissue of stress urinary incontinence rats. Int Urogynecol J (2022) 28:1–8. doi: 10.1007/s00192-022-05106-8 PMC9343271 35226143

[24] Morita E Yokoyama H Imai D Takeda R Ota A Kawai E. Aerobic exercise training with brisk walking increases intestinal bacteroides in healthy elderly women. Nutrients (2019) 11(4):868. doi: 10.3390/nu11040868 PMC6520866 30999699

[25] Lee H Lee Y Kim J An J Lee S Kong H. Modulation of the gut microbiota by metformin improves metabolic profiles in aged obese mice. Gut Microbes (2018) 9(2):155–65. doi: 10.1080/19490976.2017.1405209 PMC5989809 29157127

[26] Wexler HM. Bacteroides: the good, the bad, and the nitty-gritty. Clin Microbiol Rev (2007) 20(4):593–621. doi: 10.1128/CMR.00008-07 17934076 PMC2176045

[27] Natarajan P Punta M Kumar A Yeh AP Godzik A Aravind L. Structure and sequence analyses of bacteroides proteins BVU_4064 and BF1687 reveal presence of two novel predominantly-beta domains, predicted to be involved in lipid and cell surface interactions. BMC Bioinf (2015) 16(1):7. doi: 10.1186/s12859-014-0434-7 PMC4387736 25592227

[28] Galvão BP Weber BW Rafudeen MS Ferreira EO Patrick S Abratt VR. Identification of a collagen type I adhesin of bacteroides fragilis. PloS One (2014) 9:e91141. doi: 10.1371/journal.pone.0091141 24618940 PMC3949742

[29] Ferreira Ede O Teixeira FL Cordeiro F Araujo Lobo L Rocha ER Smith JC. The Bfp60 surface adhesin is an extracellular matrix and plasminogen protein interacting in bacteroides fragilis. Int J Med Microbiol (2013) 303:492–7. doi: 10.1016/j.ijmm.2013.06.007 PMC4022697 23850366

[30] Odamaki T Kato K Sugahara H Hashikura N Takahashi S Xiao JZ. Age-related changes in gut microbiota composition from newborn to centenarian: a cross-sectional study. BMC Microbiol (2016) 16(1):90–101. doi: 10.1186/s12866-016-0708-5 PMC4879732 27220822

[31] Chen W Liu F Ling Z Tong X Xiang C. Human intestinal lumen and mucosa-associated microbiota in patients with colorectal cancer. PloS One (2012) 7(6):e39743. doi: 10.1371/journal.pone.0039743 22761885 PMC3386193

[32] Ozato N Saito S Yamaguchi T Katashima M Tokuda I Sawada K. Blautia genus associated with visceral fat accumulation in adults 20-76 years of age. NPJ Biofilms Microbiomes (2019) 5(1):28. doi: 10.1038/s41522-019-0101-x 31602309 PMC6778088

